# Fast, cheap and sensitive: Homogenizer-based RNA extraction free method for SARS-CoV-2 detection by RT-qPCR

**DOI:** 10.3389/fcimb.2023.1074953

**Published:** 2023-03-09

**Authors:** Cristina Ramírez-Córdova, Diana Morales-Jadán, Sofía Alarcón-Salem, Alisson Sarmiento-Alvarado, María Belén Proaño, Isabel Camposano, Berenice Sarmiento-Alvarado, Mishell Bravo-Castro, Jean Franco Hidalgo-Jiménez, Dayana Coello, Ángel S. Rodríguez, Carolina Viteri-Dávila, Alexander Paolo Vallejo-Janeta, Daniela Arcos-Suárez, Miguel Angel Garcia-Bereguiain

**Affiliations:** ^1^ Laboratorio Clínico Segurilab, Quito, Ecuador; ^2^ Carrera de Ingeniería en Biotecnología, Universidad de las Américas, Quito, Ecuador; ^3^ One Health Research Group, Universidad de Las Américas, Quito, Ecuador; ^4^ Laboratorio de Investigación, Dirección General de Investigación, Universidad de Las Américas, Quito, Ecuador

**Keywords:** SARS – CoV – 2, RT-q PCR, homogenization, RNA extraction kit, COVID - 19

## Abstract

**Background:**

The SARS-CoV-2 gold standard detection method is an RT-qPCR with a previous step of viral RNA extraction from the patient sample either by using commercial automatized or manual extraction kits. This RNA extraction step is expensive and time demanding.

**Objective:**

The aim of our study was to evaluate the clinical performance of a simple SARS-CoV-2 detection protocol based on a fast and intense sample homogenization followed by direct RT-qPCR.

**Results:**

388 nasopharyngeal swabs were analyzed in this study. 222 of them tested positive for SARS-CoV-2 by the gold standard RNA extraction and RT-qPCR method, while 166 tested negative. 197 of those 222 positive samples were also positive for the homogenization protocol, yielding a sensitivity of 88.74% (95% IC; 83.83 – 92.58). 166 of those negative samples were also negative for the homogenization protocol, so the specificity obtained was 97% (95% IC; 93.11 – 99.01). For Ct values below 30, meaning a viral load of 10^3^ copies/uL, only 4 SARS-CoV-2 positive samples failed for the RNA extraction free method; for that limit of detection, the homogenizer-based method had a sensitivity of 97.92% (95% CI; 96.01 – 99.83).

**Conclusions:**

Our results show that this fast and cheap homogenization method for the SARS-CoV-2 detection by RT-qPCR is a reliable alternative of high sensitivity for potentially infectious SARS-CoV-2 positive patients. This RNA extraction free protocol would help to reduce diagnosis time and cost, and to overcome the RNA extraction kits shortage experienced during COVID-19 pandemic.

## Introduction

The world is still undergoing a pandemic called COVID-19 that has affected millions of people since the initial outbreak in December 2019. This is caused by the SARS-CoV-2 virus, that belongs to the β-coronavirus family that causes severe acute respiratory syndrome (SARS). This syndrome affects the human body systemically, through the evasion of various control points of the innate immune system, causing cases ranging from asymptomatic to severe inflammation episodes (Ortiz-Prado et al., 2020). Up to October 2022, more that 625 million COVID-19 cases and more that 6.5 million of COVID-19 related deaths have been reported (Center for Systems Science and Engineering, 2022).

Several molecular methods allow the identification of SARS-CoV-2, but the gold standard is still the RT-qPCR from nasopharyngeal samples, where the viral detection requires a correct extraction of a viable genetic material (Freire-Paspuel et al., 2020). There are several methods of RNA extraction such as chemical and mechanical, as well as commercial kits that assure an optimal RNA extraction (Fomsgaard and Rosenstierne, 2020). All these extraction methods are time consuming and causes a delay in clinical diagnosis. Moreover, this RNA extraction step increases the cost of the analysis by using more reagents and disposable supplies (Morehouse et al., 2021). One of the most conventional nucleic acid extraction methods is isolation by solid chromatography, commonly known as ARN extraction kit by columns, which traps genomic material by resins charged positively, followed by elution steps that yield a high-quality pure sample.

Several alternatives to conventional RNA extraction kits have been proposed for SARS-CoV-2 detection. Those alternatives methods combine heat shock treatment and/or lysis buffers, and the sensitivity reported varies from 77,5% to 100% (Bruno et al., 2021). Moreover, osmotic and mechanical lysis has been previously proposed as an alternative to RNA extraction for viral nucleic acid detection (Moore et al., 2008). However, to the best of our knowledge, this fast and cheap method has not yet been tested for SARS-CoV-2 diagnosis. Therefore, the aim of this study was to evaluate the clinical performance of a simple SARS-CoV-2 detection protocol based in homogenization with a bead mill followed by direct RT-qPCR.

## Materials and methods

### Sample collection

**Figure 1:**
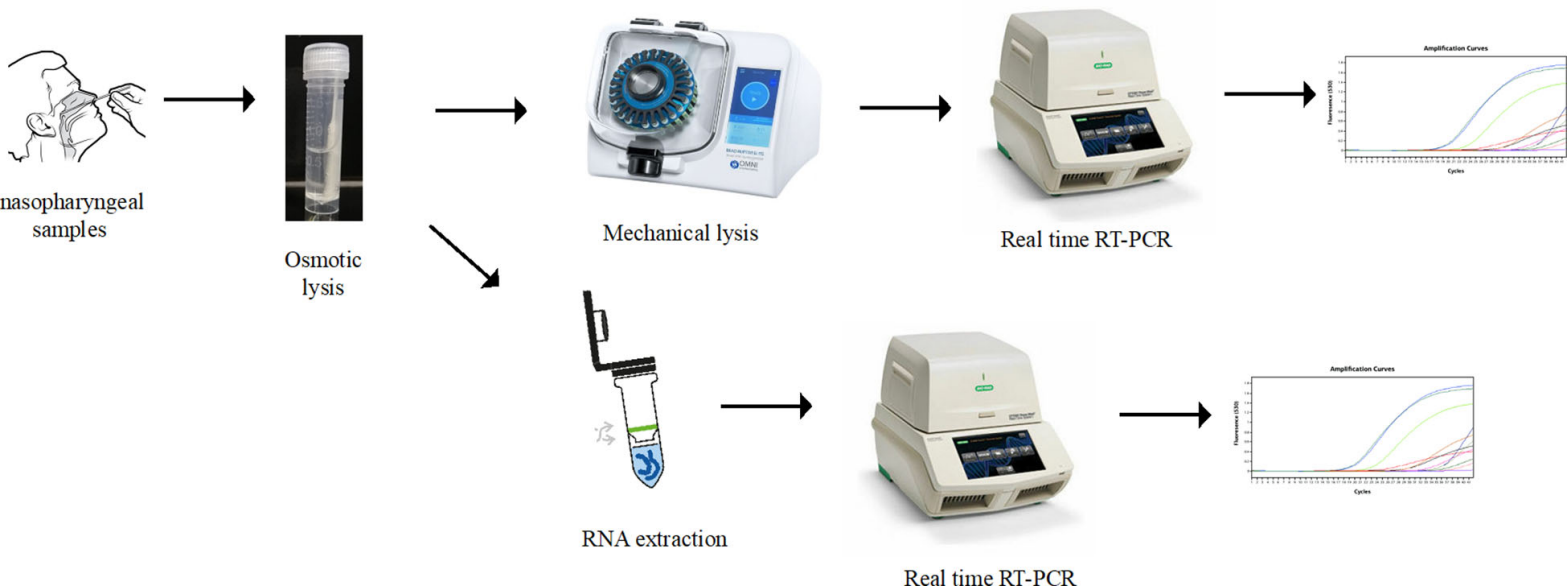
Flow chart for nasopharyngeal samples processing for SARS-CoV-2 detection by RT-qPCR for the regular RNA extraction method and for the RNA extraction free protocol based on mechanical lysis with a homogenizer.

nasopharyngeal samples were collected in plastic tubes containing 2mL of ultrapure water. A total of 388 nasopharyngeal samples were included in the study (**Supplementary Table 1**). Those samples were randomly selected as the SARS-CoV-2 positivity rate was really high during the period this study was done. Those samples followed two sequential SARS-CoV-2 diagnosis protocols as illustrated in **Figure 1**: a) the standard RNA extraction using commercial column kits; b) a mechanical lysis using a homogenizer without RNA extraction.

### Mechanical lysis by sample homogenization

Homogenization was performed in the Bead Ruptor Elite equipment (OMINI International, USA) whose operation is based on the vigorously shaking of the vials that contains sample at a set speed and time to lyse cells. Despite being a bead mill homogenizer, no beads are used in this processing. The homogenization program is regulated at 4 m/s for 30 seconds.

*RNA extraction using the Virus DNA/RNA Purification kit (Biocomma, China).* RNA extraction was carried out using this commercial kit by following the manufacturer’s manual.

### SARS-CoV-2 detection by RT-qPCR

Both the homogenized samples and the RNA extractions were processed for SARS-CoV-2 detection by RT-qPCR as we have previously reported (Freire-Paspuel and Garcia-Bereguiain, 2021a; Freire-Paspuel and Garcia-Bereguiain, 2021b; Freire-Paspuel and Garcia-Bereguiain, 2021c; Freire-Paspuel et al., 2021; Santander-Gordon et al., 2021; Freire-Paspuel et al., 2022; Rodriguez-Paredes et al., 2022). The RT-qPCR was performed in a the CFX96 (Biorad, USA) equipment using the commercial kit ECUGEN SARS-CoV-2 RT-qPCR (Starnewcorp-UDLA, Ecuador), a triplex assay based on the CDC protocol that includes the viral targets N1 and N2, and the RNase P gene as a control for RNA extraction quality. According to the manufacturer, its sensitivity is 97.7% and specificity 100% (Freire-Paspuel and Garcia-Bereguiain, 2021a; Freire-Paspuel et al., 2022). As we could not have access to a BSL3 lab for SARS-CoV-2 cultures, we could not address sample tittering with known concentration of SARS-CoV-2 viral particles. So, for viral loads calculation, the 2019-nCoV N positive control (IDT, USA) was used, provided at 200.000 genome equivalents/uL. This positive control is a plasmid including N1 and N2 viral gene targets sequences and it is a SARS-CoV-2 positive control recommended by CDC guidelines (Freire-Paspuel and Garcia-Bereguiain, 2021a; Freire-Paspuel and Garcia-Bereguiain, 2021b; Freire-Paspuel et al., 2022). Serial dilutions of the positive control were included in each set of samples RT-qPCR running, so an internal calibration curve with triplicates of known concentrations of genomic SARS-CoV-2 material was always available. A regression analysis was made for each of those calibration curves taking RT-qPCR Ct values for N1 and N2 targets and viral genomic material concentrations as variables; the equation obtained was used for viral load calculations for each set of clinical, finally expressed of an average of the values for N1 and N2 targets. Regression coefficients over 0.99 were obtained for the viral load calibration curves. The viral loads are expressed in copies/uL of RNA extraction, and the conversion factor for copies/mL of sample media is 200 in our experimental conditions.

### Statistical analysis

The sensitivity, specificity, positive predictive value, and negative predictive value were calculated with confidence level of 95%. The t-Student was used to compare the Ct values obtained by each of those values for each protocol. Kappa-Cohen coefficient was calculated to measure the degree of agreement between two methods. All statistical analysis was carried out using SPSS Statistics 23 software.

## Results

### Clinical performance of the homogenizer-based RNA extraction free method compared to the standard column based RNA extraction

A total of 388 nasopharyngeal samples were included in the study, of which 222 were positive and 166 were negative for SARS-CoV-2 using the column extraction method followed by RT-qPCR. 197 out of 222 SARS-CoV-2 positive samples tested also positive with the alternative homogenizer method. Moreover, 161 SARS-CoV-2 negative samples were also negative for the mechanical lysis method without RNA extraction (See **Supplementary Table 1**). So, the overall sensitivity was 88.74% (95% IC; 83.83 – 92.58) and specificity was 97% (95% IC; 93.11 – 99.01). Also, Positive Likelihood Ratio was 29.46 (95% CI 12.41 - 69.94); and Negative Likelihood Ratio was 0.12 (95% CI 0.08 - 0.17). Considering the SARS-CoV-2 infection rate obtained in the sample set included in this study, a positive predictive value of 97.52% (IC 95%; 93.3 – 99.6) and a negative predictive value of 86.56% (IC 95%; 81.6 – 91.5) was obtained. The Kappa Cohen value for the comparison between the RNA extraction method and the homogenization method was 0.844, which means a “very good” agreement between both protocols.

In **Table 1**, the values of the clinical performance parameters for different Ct values thresholds are detailed. For Ct < 25, the sensitivity was 100%. For Ct < 30, the sensitivity was 97.92% (IC 95%; 96.01 – 99.83).

**Table 1:** Evaluation of the clinical performance of the RNA extraction free homogenization-based method for SARS-CoV-2 detection.

Cycle Threshold (Ct) N1	N	Sensitivity (%)	Specificity (%)	% NPV
**≤ 25**	168/168	100	N: 161/166; 96,98 (94.4 – 99.6)	100
**≤ 30**	189/193	97.92 (96.01 – 99.83)		97.57 (95.22 – 99.92)
**< 40**	197/222	88.74 (84.58 – 92.9)		86.55 (81.6 – 91.45)

Sensitivity, Specificity and Negative Predictive Values (NPV) for different cycle threshold values by RT-qPCR are presented with 95% confidence interval.

### Analytical sensitivity of the homogenizer-based RNA extraction free method compared to the standard column based RNA extraction

**Figure 2:**
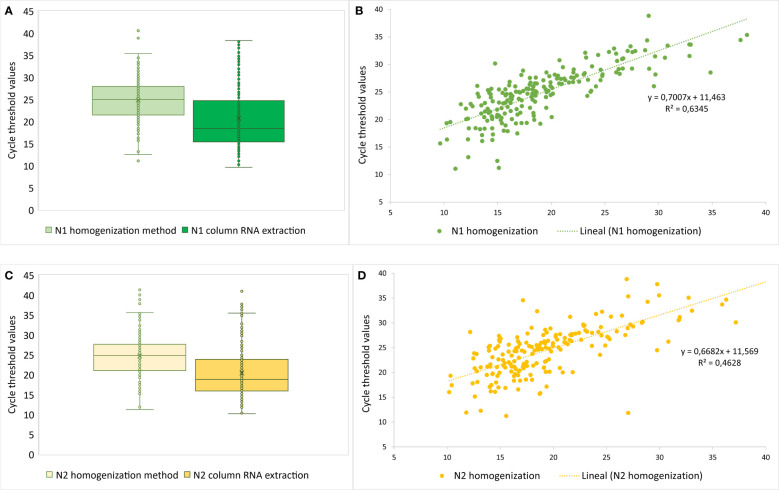
**(A-C)** Average Ct values obtained for N1 and N2 genes using homogenization method vs column RNA extraction (gold standard); **(B-D)** Linear plots for Ct values for N1 and N2 genes for RNA extraction versus homogenization method.

The analytical sensitivity for the homogenizer-based RNA extraction free method was addressed by comparing the Ct values obtained for the viral N1 and N2 targets (**Figure 2**). The mean Ct value for N1 was 21.42 ± 7.10, and for N2 was 19.93 ± 5.8, with the column based RNA extraction protocol. The mean Ct value for N1 was 25.27 ± 4.81, and for N2 was 24.54 ± 5.25, with the homogenizer-based RNA extraction free method. These differences between both methods for the mean N1 and N2 Ct values were statistically significant (p<0.001). In **Figure 2B**, the linear regression analysis for the N1 and N2 Ct values for both protocols used is displayed, showing a significant linear adjustment with R^2^ values of 0.63 and 0.46.

In **Supplementary Table 2**, the Ct values and viral loads (RNA copies/uL) for the 25 false negative samples with the RNA extraction free protocol by homogenization are detailed. Only 4 of those false negative samples have N1 Ct values below 30, meaning viral loads over 10^3^ copies/uL. The sensitivity for the homogenizer-based RNA extraction free method for SARS-CoV-2 detection by RT-qPCR was 97.92% (189/193) for a Limit of Detection (LoD) of 10^3^ copies/uL.

## Discussion

Our results endorse that the RNA extraction free homogenization methodology for SARS-CoV-2 detection by RT-qPCR presented in this study is a reliable alternative to the classic column based RNA extraction protocols. We obtained an overall high sensitivity (88.74%) and specificity (97%), but also for Ct values below 30 or viral loads of 10^3^ copies/uL of RNA extraction (200,000 copies/mL of sample media) the sensitivity was as high as 97.92%. Considering the viral loads associated to SARS-CoV-2 transmission (Wölfel et al., 2020), this protocol for SARS-CoV-2 detection by RT-qPCR without RNA extraction would potentially detect all the infectious individuals.

In a previous study from our lab, we revised the sensitivity of heat shock based RNA extraction free methods compared to the classic RNA extraction protocols for SARS-CoV-2 detection by RT-qPCR; the sensitivity values for those protocols vary from 54% to 97% depending on the reports. Also, a direct sample to PCR assay protocol without any treatment or RNA extraction have been reported to achieve a sensitivity of 92% (Bruce et al., 2020). Although our protocol includes a step of sample collection in ultra pure water, we could not get a high sensitivity for osmotic lysis, even for samples with Ct values lower than 25; so we decided to include a homogenization step following the osmotic lysis. Our mechanical lysis by homogenization protocol accomplished even a better sensitivity than those other heat shock based methods, up to 97.92% for infectious viral loads. On the other hand, this method has two drawbacks: 1) individuals in the early phase of the infection could be reported as false negative due to the the reduced sensitivity; 2) we cannot totally rule out that the small reduction of specificity could be attributed to cross contamination due to the vigorous homogenization step, although this issue may also happen with the standard RNA extraction method.

The development of sensitive RNA extraction free protocols for SARS-CoV-2 detection by RT-qPCR represents a reliable alternative to overcome the main challenges experienced during COVID-19 pandemics, specially at LMICs: 1) Cost reduction of testing by suppressing the RNA extraction with commercial kits; 2) Avoiding RNA extraction kits supply shortage as it was experienced during COVID-19 pandemics; 3) Speeding up the sample processing as RNA extraction methods are time consuming, and testing demands under pandemics scenarios are extremely high.

In conclusion, while keeping the described drawbacks of this method in mind, this fast, cheap and sensitive homogenizer-based RNA extraction free method for SARS-CoV-2 detection by RT-qPCR would support a more equitable COVID-19 testing capacities for public health systems at LMICs. Moreover, it is an important lesson for future pandemics: more flexible protocols that keep a high sensitivity will allow to have a more equity for testing in future pandemics.

## Data availability statement

The original contributions presented in the study are included in the article/**Supplementary Material**. Further inquiries can be directed to the corresponding authors.

## Ethics statement

The paired samples used for the homogenization protocol were the leftover of the samples collected for routine SARS-CoV-2 diagnosis. Nevertheless, this work is included in a study that was approved by the IRB from the Dirección Nacional de Inteligencia de la Salud (Ministerio de Salud Publica, Ecuador) under the code 008-2020.

## Author contributions

All the authors contributed to experimental design and lab work. CR-C, DM-J and MG-B wrote the first draft of the manuscript. MG-B wrote the final version. All authors contributed to the article and approved the submitted version.
