# Supplementary material for: Fast, cheap and sensitive: Homogenizer-based RNA extraction free method for SARS-CoV-2 detection by RT-qPCR
Source: Front Cell Infect Microbiol. 2023 Mar 9;13:1074953. doi: 10.3389/fcimb.2023.1074953 (PMC10035754; doi:10.3389/fcimb.2023.1074953)
Supplement: Supplementary file 1 [file Table_1.docx]

**Supplementary Table 1**. Nasopharyngeal samples with Ct values for N1, N2 and RNase P gene and viral loads for SARS-CoV-2 positives samples with homogenization and RNA extraction methods. Viral loads are detailed on copies/uL of RNA extraction solution.

|  | | Homogenization method | | | | Column RNA Extraction | | | | |
| --- | --- | --- | --- | --- | --- | --- | --- | --- | --- | --- |
| N | **Code** | **N1 Ct** | **N2 Ct** | **RP Ct** | **Result** | **N1 Ct** | **N2 Ct** | **RP Ct** | **Result** | **Viral Load (copies/uL)** |
| 1 | 1 | 25,35 | 24,82 | 26,14 | Positive | 14,09 | 14,54 | 32,27 | Positive | 1,62E+07 |
| 2 | 2 | 24,91 | 25,35 | 25,14 | Positive | 17,08 | 17,71 | 22,85 | Positive | 2,51E+06 |
| 3 | 3 | 17,48 | 18,56 | 28,04 | Positive | 16,83 | 17,42 | 21,21 | Positive | 2,94E+06 |
| 4 | 4 | 35,37 | NA | 28,76 | Positive | NA | NA | 23,66 | Negative |  |
| 5 | 5 | 20,32 | 20,21 | 26,58 | Positive | 15,35 | 16,03 | 33,53 | Positive | 7,38E+06 |
| 6 | 6 | 37,01 | NA | 29,89 | Positive | NA | NA | 27,57 | Negative |  |
| 7 | 7 | 33,60 | NA | 29,81 | Positive | 33 | 35,44 | 27,81 | Positive | 1,24E+02 |
| 8 | 8 | 34,44 | NA | 29,05 | Positive | 37,64 | NA | 24,43 | Positive | 6,90E+00 |
| 9 | 9 | 22,13 | 22,66 | 27,12 | Positive | 18,01 | 19,57 | 24,22 | Positive | 1,41E+06 |
| 10 | 10 | 24,38 | 25,79 | 23,95 | Positive | 15,73 | 16,82 | 20,79 | Positive | 5,83E+06 |
| 11 | 11 | 32,45 | NA | 24,83 | Positive | 27,11 | 27,96 | 23,6 | Positive | 4,87E+03 |
| 12 | 12 | 15,66 | 16,06 | 28,23 | Positive | 9,63 | 10,18 | NA | Positive | 2,60E+08 |
| 13 | 13 | 25,00 | 26,03 | 22,99 | Positive | 16,98 | 17,45 | 19,68 | Positive | 2,68E+06 |
| 14 | 14 | 32,29 | 40,01 | 24,58 | Positive | 25,38 | 26,13 | 21,57 | Positive | 1,43E+04 |
| 15 | 15 | 22,79 | 23,88 | 25,90 | Positive | 11,56 | 12,53 | NA | Positive | 7,82E+07 |
| 16 | 16 | 26,81 | 28,25 | 26,26 | Positive | 22,02 | 23,16 | 22,37 | Positive | 1,16E+05 |
| 17 | 17 | 26,19 | 27,10 | 24,34 | Positive | 16,06 | 16,58 | 23,85 | Positive | 4,74E+06 |
| 18 | 18 | 22,83 | 22,86 | 23,42 | Positive | 12,47 | 12,45 | 21,98 | Positive | 4,44E+07 |
| 19 | 20 | 16,26 | 16,91 | 28,28 | Positive | 14,5 | 15,48 | 29,81 | Positive | 1,25E+07 |
| 20 | 21 | 18,43 | 19,53 | 24,79 | Positive | 12,94 | 13,72 | 26,39 | Positive | 3,31E+07 |
| 21 | 22 | 21,09 | 19,45 | 26,22 | Positive | 15,65 | 16,67 | NA | Positive | 6,13E+06 |
| 22 | 23 | 25,22 | 23,56 | 27,00 | Positive | 23,72 | 24,39 | 23,2 | Positive | 4,02E+04 |
| 23 | 24 | 22,45 | 21,88 | 26,61 | Positive | 17,25 | 18,41 | 24,81 | Positive | 2,26E+06 |
| 24 | 27 | 21,24 | 15,70 | 32,42 | Positive | 17,11 | 18,7 | 31,72 | Positive | 2,47E+06 |
| 25 | 28 | 18,26 | 17,46 | 22,92 | Positive | 13,34 | 14,15 | 20,26 | Positive | 2,58E+07 |
| 26 | 29 | 19,94 | 20,13 | 23,02 | Positive | 18,41 | 19,27 | 21,78 | Positive | 1,10E+06 |
| 27 | 41 | 28,59 | 11,87 | 25,64 | Positive | 26,06 | 27,04 | 21,38 | Positive | 9,36E+03 |
| 28 | 42 | 31,24 | 30,63 | 28,69 | Positive | 30,6 | 31,81 | 28,43 | Positive | 5,54E+02 |
| 29 | 43 | 22,29 | 21,14 | 22,52 | Positive | 15,93 | 16,32 | 19,19 | Positive | 5,15E+06 |
| 30 | 44 | 18,40 | 18,09 | 26,40 | Positive | 12,3 | 12,84 | 29,71 | Positive | 4,93E+07 |
| 31 | 45 | 19,31 | 19,35 | 25,59 | Positive | 10,23 | 10,32 | 29,97 | Positive | 1,79E+08 |
| 32 | 46 | 21,94 | 22,66 | 26,00 | Positive | 14,06 | 14,83 | 24,4 | Positive | 1,65E+07 |
| 33 | 47 | 24,68 | 24,81 | 25,01 | Positive | 18,23 | 19,6 | 23,28 | Positive | 1,23E+06 |
| 34 | 48 | 24,59 | 25,30 | 26,12 | Positive | 14,35 | 14,79 | 27,99 | Positive | 1,38E+07 |
| 35 | 51 | 22,56 | 23,10 | 27,48 | Positive | 13,47 | 14,25 | 28,38 | Positive | 2,38E+07 |
| 36 | 52 | 26,01 | 24,70 | 26,74 | Positive | 18,33 | 18,66 | 23,42 | Positive | 1,15E+06 |
| 37 | 53 | 27,45 | 27,83 | 24,69 | Positive | 21,21 | 22,06 | 21,84 | Positive | 1,92E+05 |
| 38 | 59 | 21,20 | 21,07 | 25,17 | Positive | 14,16 | 14,68 | 27,62 | Positive | 1,55E+07 |
| 39 | 60 | 20,45 | 20,15 | 23,78 | Positive | 20,04 | 19,64 | 20,45 | Positive | 3,98E+05 |
| 40 | 61 | 24,45 | 24,26 | 25,84 | Positive | 19,94 | 19,78 | 21,88 | Positive | 4,23E+05 |
| 41 | 62 | 20,89 | 20,27 | 26,89 | Positive | 16,84 | 16,73 | 26,62 | Positive | 2,92E+06 |
| 42 | 63 | 19,38 | 19,52 | 30,28 | Positive | 17,71 | 17,84 | 26,19 | Positive | 1,70E+06 |
| 43 | 64 | 24,14 | 25,11 | 24,24 | Positive | 13,24 | 13,91 | 21,95 | Positive | 2,75E+07 |
| 44 | 65 | 26,11 | 23,73 | 22,78 | Positive | 13,09 | 12,76 | 21,05 | Positive | 3,02E+07 |
| 45 | 66 | 26,76 | 24,61 | 27,34 | Positive | 19,92 | 20,67 | 24,71 | Positive | 4,29E+05 |
| 46 | 67 | 24,35 | 20,96 | 26,00 | Positive | 18,53 | 19,09 | 21,79 | Positive | 1,02E+06 |
| 47 | 68 | 23,03 | 21,14 | 24,26 | Positive | 16,21 | 17,12 | 24,09 | Positive | 4,32E+06 |
| 48 | 69 | 28,19 | 25,37 | 26,48 | Positive | 23,02 | 23,62 | 21,2 | Positive | 6,22E+04 |
| 49 | 70 | 22,41 | 21,05 | 22,40 | Positive | 12,65 | 13,16 | 19,6 | Positive | 3,97E+07 |
| 50 | 71 | 23,71 | 20,85 | 26,34 | Positive | 16,31 | 17,17 | 19,91 | Positive | 4,06E+06 |
| 51 | 72 | 21,98 | 15,17 | 28,23 | Positive | 12,04 | 12,6 | NA | Positive | 5,80E+07 |
| 52 | 73 | 26,48 | 26,56 | 24,57 | Positive | 20,98 | 21,53 | 21,99 | Positive | 2,22E+05 |
| 53 | 74 | 20,85 | 21,66 | 21,58 | Positive | 14,96 | 15,96 | 20,19 | Positive | 9,41E+06 |
| 54 | 75 | 24,99 | 25,04 | 24,16 | Positive | 18,61 | 19,27 | 21,47 | Positive | 9,69E+05 |
| 55 | 76 | 29,16 | 27,57 | 25,01 | Positive | 26,27 | 26,77 | 22,23 | Positive | 8,21E+03 |
| 56 | 77 | 20,47 | 19,30 | 23,08 | Positive | 14,93 | 14,73 | 28,02 | Positive | 9,59E+06 |
| 57 | 78 | 20,60 | 21,62 | 23,79 | Positive | 14,43 | 14,06 | 23,07 | Positive | 1,31E+07 |
| 58 | 79 | 33,28 | 41,26 | 26,40 | Positive | 27,39 | 27,54 | 23,7 | Positive | 4,09E+03 |
| 59 | 80 | 25,20 | 25,36 | 26,30 | Positive | 19,73 | 19,94 | 22,33 | Positive | 4,83E+05 |
| 60 | 81 | 29,23 | 32,46 | 24,57 | Positive | 29,12 | 33,04 | 22,14 | Positive | 1,39E+03 |
| 61 | 82 | 18,91 | 19,69 | 25,10 | Positive | 15,73 | 16,38 | 24,34 | Positive | 5,83E+06 |
| 62 | 83 | 23,03 | 20,73 | 24,28 | Positive | 16,19 | 16,42 | 25,65 | Positive | 4,38E+06 |
| 63 | 84 | 29,04 | 31,26 | 26,21 | Positive | 24,6 | 25,41 | 23,2 | Positive | 2,32E+04 |
| 64 | 85 | 22,14 | 20,02 | 26,84 | Positive | 19,53 | 20,88 | 25,6 | Positive | 5,47E+05 |
| 65 | 86 | 23,08 | 24,33 | 22,84 | Positive | 15,11 | 16,33 | NA | Positive | 8,57E+06 |
| 66 | 87 | 33,63 | 35,09 | 30,35 | Positive | 32,87 | 32,73 | 26,32 | Positive | 1,35E+02 |
| 67 | 88 | 28,90 | 34,57 | 26,13 | Positive | 17,36 | 17,12 | 22,7 | Positive | 2,11E+06 |
| 68 | 89 | 31,71 | 32,25 | 25,89 | Positive | 24,36 | 24,58 | 20,79 | Positive | 2,70E+04 |
| 69 | 90 | 16,37 | 17,44 | 24,62 | Positive | 10,26 | 10,43 | 28,36 | Positive | 1,76E+08 |
| 70 | 91 | 23,78 | 24,13 | 27,73 | Positive | NA | NA | 34,68 | Negative |  |
| 71 | 92 | 21,54 | 21,32 | 26,95 | Positive | 16,21 | 17,5 | NA | Positive | 4,32E+06 |
| 72 | 93 | 26,55 | 26,13 | 25,19 | Positive | 20,73 | 21,03 | 22,54 | Positive | 2,59E+05 |
| 73 | 94 | 25,5 | 25,6 | 28,05 | Positive | 15,72 | 15,8 | NA | Positive | 5,86E+06 |
| 74 | 95 | 29,27 | 30,04 | 25,71 | Positive | 27,52 | 28,33 | 24,65 | Positive | 3,77E+03 |
| 75 | 96 | 25,45 | 26,25 | 27,56 | Positive | 18,24 | 19,14 | 31,32 | Positive | 1,22E+06 |
| 76 | 97 | 21,78 | 21,89 | 24,95 | Positive | 14,95 | 15,39 | 25,02 | Positive | 9,47E+06 |
| 77 | 98 | 16,09 | 16,23 | 24,76 | Positive | 13,55 | 14,1 | 40,54 | Positive | 2,27E+07 |
| 78 | 99 | 24,67 | 24,47 | 27,07 | Positive | 18,32 | 18,22 | 30,89 | Positive | 1,16E+06 |
| 79 | 100 | 27,17 | 27,23 | 25,03 | Positive | 24,59 | 25,07 | 20,64 | Positive | 2,34E+04 |
| 80 | 101 | 23,49 | 23,76 | 25,93 | Positive | 17,24 | 18,02 | 21,52 | Positive | 2,28E+06 |
| 81 | 102 | 21,09 | 21,18 | 25,24 | Positive | 13,45 | 14,03 | 30,41 | Positive | 2,41E+07 |
| 82 | 103 | 28,55 | 29,51 | 25,09 | Positive | 22,05 | 22,92 | 21,74 | Positive | 1,14E+05 |
| 83 | 104 | 29,26 | 29,3 | 27,41 | Positive | 26,65 | 27,49 | 23,13 | Positive | 6,48E+03 |
| 84 | 105 | 27,66 | 28,01 | 30,1 | Positive | 21,85 | 22,4 | 27,07 | Positive | 1,29E+05 |
| 85 | 106 | 28,08 | 29,01 | 27 | Positive | 17,2 | 17,94 | 25,64 | Positive | 2,33E+06 |
| 86 | 107 | 27,87 | 27,55 | 23,74 | Positive | 19,81 | 20,22 | 20,03 | Positive | 4,59E+05 |
| 87 | 108 | 22,92 | 22,27 | 25,91 | Positive | 15,51 | 16,32 | 21,59 | Positive | 6,68E+06 |
| 88 | 109 | 26,34 | 26 | 29,76 | Positive | 17,98 | 18,82 | 31,97 | Positive | 1,44E+06 |
| 89 | 110 | 19,8 | 19,95 | 25,27 | Positive | 14,03 | 14,92 | 27,33 | Positive | 1,68E+07 |
| 90 | 111 | 28 | 28,5 | 26,9 | Positive | 22,37 | 23,01 | 24,88 | Positive | 9,32E+04 |
| 91 | 112 | 24 | 27,38 | 24,88 | Positive | 14,36 | 14,87 | 26,38 | Positive | 1,37E+07 |
| 92 | 113 | 11,22 | 11,23 | 22,53 | Positive | 15,1 | 15,55 | 20,17 | Positive | 8,63E+06 |
| 93 | 114 | 24,06 | 25,07 | 29,22 | Positive | 19,49 | 20,47 | 25,5 | Positive | 5,60E+05 |
| 94 | 115 | 24,13 | 24,74 | 24,12 | Positive | 18,15 | 18,9 | 18,22 | Positive | 1,29E+06 |
| 95 | 116 | 30,64 | 35,34 | 27,77 | Positive | 26,14 | 27,04 | 23,13 | Positive | 8,91E+03 |
| 96 | 117 | 21,42 | 22,52 | 25,42 | Positive | 14,29 | 15,26 | 31,09 | Positive | 1,43E+07 |
| 97 | 118 | 25,16 | 27,57 | 26,89 | Positive | 18,51 | 19,05 | 23,72 | Positive | 1,03E+06 |
| 98 | 119 | 26,88 | NA | 25,46 | Positive | 22,6 | 23,55 | 20,17 | Positive | 8,08E+04 |
| 99 | 120 | 17,98 | 19,28 | 30,97 | Positive | 15,56 | 16,29 | 23,4 | Positive | 6,48E+06 |
| 100 | 121 | 11,06 | 11,94 | NA | Positive | 11,06 | 11,79 | 29,85 | Positive | 1,07E+08 |
| 101 | 122 | 12,48 | 15,83 | 35,37 | Positive | 14,96 | 18,77 | NA | Positive | 9,41E+06 |
| 102 | 123 | 28,18 | 31,16 | 27,72 | Positive | 29,71 | 31,89 | 22,47 | Positive | 9,64E+02 |
| 103 | 124 | 32,6 | 26,23 | 27,56 | Positive | 28,75 | 30,82 | 25,13 | Positive | 1,75E+03 |
| 104 | 125 | 34,68 | 31,96 | 29,15 | Positive | NA | NA | 26,58 | Negative |  |
| 105 | 126 | 20,05 | 18,35 | 23,97 | Positive | 16,32 | 16,85 | 23,53 | Positive | 4,04E+06 |
| 106 | 127 | 31,26 | 28,21 | 25,39 | Positive | 23,24 | 24,22 | 23,07 | Positive | 5,42E+04 |
| 107 | 128 | 24,02 | 21,71 | 23,26 | Positive | 16,21 | 17,19 | 22,86 | Positive | 4,32E+06 |
| 108 | 129 | 25,61 | 25,5 | 27,2 | Positive | 17,7 | 18,41 | 24,56 | Positive | 1,71E+06 |
| 109 | 130 | 28,42 | 28,01 | 26,68 | Positive | 23,12 | 23,63 | 23,7 | Positive | 5,84E+04 |
| 110 | 131 | 18,34 | 16,12 | 27,25 | Positive | 13,77 | 14,51 | 23,65 | Positive | 1,98E+07 |
| 111 | 132 | 25,02 | 26,55 | 21,67 | Positive | 14,19 | 14,88 | 21,1 | Positive | 1,52E+07 |
| 112 | 133 | 28,54 | 30,11 | 21,9 | Positive | 34,84 | 37,17 | 25,61 | Positive | 3,95E+01 |
| 113 | 134 | 29,65 | 29,17 | 24,51 | Positive | 23,79 | 24,46 | 24 | Positive | 3,85E+04 |
| 114 | 135 | 32,12 | 31,8 | 21,51 | Positive | 23,17 | 24 | 23,04 | Positive | 5,66E+04 |
| 115 | 136 | 25,54 | 24,94 | 17,74 | Positive | 17,86 | 18,52 | 22,68 | Positive | 1,55E+06 |
| 116 | 137 | 35,35 | 40,2 | 25,23 | Positive | 38,25 | N/A | 25,45 | Positive | 4,72E+00 |
| 117 | 138 | 32,49 | 36,97 | 24,17 | Positive | NA | N/A | 25,02 | Negative |  |
| 118 | 139 | 26,02 | 28,29 | 22,72 | Positive | 24,16 | 25,96 | 25,74 | Positive | 3,06E+04 |
| 119 | 140 | 32,25 | 30,23 | 22,72 | Positive | 27,55 | 28,4 | 25,02 | Positive | 3,70E+03 |
| 120 | 141 | 16,41 | 17,82 | 26,63 | Positive | 12,23 | 12,39 | 29,29 | Positive | 5,15E+07 |
| 121 | 142 | 27,56 | 29,39 | 26,18 | Positive | 22,09 | 22,86 | 23,92 | Positive | 1,11E+05 |
| 122 | 143 | 26,03 | 27,86 | 23,53 | Positive | 16,63 | 17,55 | 21,61 | Positive | 3,33E+06 |
| 123 | 144 | 34,38 | 37,8 | 24,44 | Positive | 28,94 | 29,77 | 23,72 | Positive | 1,56E+03 |
| 124 | 145 | 17,3 | 17 | 27,54 | Positive | 14,54 | 14,82 | 34,37 | Positive | 1,22E+07 |
| 125 | 146 | 26,07 | 27,71 | 25,05 | Positive | 19,92 | 20,62 | 24,27 | Positive | 4,29E+05 |
| 126 | 147 | 23,05 | 23,45 | 26,22 | Positive | 15,72 | 16,06 | 37,46 | Positive | 5,86E+06 |
| 127 | 148 | 24,3 | 25,45 | 28,4 | Positive | 23,35 | 24,88 | 28,02 | Positive | 5,06E+04 |
| 128 | 149 | 24,64 | 24,91 | 26,37 | Positive | 16,21 | 16,48 | 29,06 | Positive | 4,32E+06 |
| 129 | 150 | 21,44 | 21,19 | 24,13 | Positive | 13,81 | 13,78 | 29,21 | Positive | 1,93E+07 |
| 130 | 151 | 25,5 | 26,63 | 25,08 | Positive | 19,47 | 20,35 | 22,08 | Positive | 5,67E+05 |
| 131 | 152 | 27,13 | 28,89 | 26 | Positive | 19,26 | 19,72 | 22,34 | Positive | 6,47E+05 |
| 132 | 153 | 24,69 | 25,39 | 24,1 | Positive | 13,12 | 14,01 | 21,96 | Positive | 2,96E+07 |
| 133 | 154 | 27,94 | 27,05 | 26,05 | Positive | 24,75 | 25,18 | 23,46 | Positive | 2,12E+04 |
| 134 | 155 | 31,54 | 34,72 | 26,88 | Positive | 32,88 | 36,26 | 24,25 | Positive | 1,34E+02 |
| 135 | 156 | 24,78 | 26,21 | 23,37 | Positive | 14,23 | 14,95 | 20,32 | Positive | 1,48E+07 |
| 136 | 157 | 32,42 | 34,27 | 26,5 | Positive | 27,83 | 28,87 | 22,6 | Positive | 3,11E+03 |
| 137 | 158 | 29,11 | 29,65 | 25,77 | Positive | 22,08 | 22,8 | 22,78 | Positive | 1,12E+05 |
| 138 | 159 | NA | NA | 25,14 | Negative | NA | NA | 23,98 | Negative |  |
| 139 | 160 | NA | NA | 25,38 | Negative | NA | NA | 21,56 | Negative |  |
| 140 | 161 | NA | NA | 25,65 | Negative | NA | NA | 25,21 | Negative |  |
| 141 | 162 | NA | NA | 26,08 | Negative | 36,9 | 37,02 | 22,34 | Positive | 1,09E+01 |
| 142 | 163 | NA | NA | 22,16 | Negative | NA | NA | 22,46 | Negative |  |
| 143 | 164 | NA | NA | 27,05 | Negative | 34,01 | 37,21 | 28,12 | Positive | 6,62E+01 |
| 144 | 165 | NA | NA | 24,65 | Negative | 37,58 | N/A | 20,72 | Positive | 7,17E+00 |
| 145 | 166 | NA | NA | 27,5 | Negative | NA | N/A | 25,65 | Negative |  |
| 146 | 167 | NA | NA | 27,52 | Negative | NA | N/A | 24,42 | Negative |  |
| 147 | 168 | NA | NA | 24,67 | Negative | NA | NA | 23,29 | Negative |  |
| 148 | 169 | NA | NA | 27,1 | Negative | N/A | N/A | 23,25 | Negative |  |
| 149 | 170 | NA | NA | 27,03 | Negative | 33,64 | N/A | 24,59 | Positive | 8,34E+01 |
| 150 | 171 | NA | NA | 22,1 | Negative | N/A | N/A | 22,52 | Negative |  |
| 151 | 172 | NA | NA | 25,64 | Negative | N/A | N/A | 25,36 | Negative |  |
| 152 | 173 | NA | NA | 24,94 | Negative | N/A | N/A | 23,08 | Negative |  |
| 153 | 174 | NA | NA | 27,33 | Negative | 40,14 | N/A | 27,17 | Negative | 1,45E+00 |
| 154 | 175 | NA | NA | 25,66 | Negative | N/A | N/A | 23,38 | Negative |  |
| 155 | 176 | NA | NA | 25,79 | Negative | N/A | N/A | 25,09 | Negative |  |
| 156 | 177 | NA | NA | 26,12 | Negative | NA | NA | 21,3 | Negative |  |
| 157 | 178 | NA | NA | 24,17 | Negative | NA | NA | 23,67 | Negative |  |
| 158 | 179 | NA | NA | 26,07 | Negative | N/A | N/A | 23,87 | Negative |  |
| 159 | 180 | NA | NA | 23,01 | Negative | NA | NA | 21,31 | Negative |  |
| 160 | 181 | NA | NA | 27,13 | Negative | 38,03 | N/A | 24,9 | Positive | 5,41E+00 |
| 161 | 182 | NA | NA | 24,04 | Negative | 33,53 | NA | 22,26 | Positive | 8,93E+01 |
| 162 | 183 | NA | NA | 24,17 | Negative | NA | NA | 24,78 | Negative |  |
| 163 | 184 | NA | NA | 26,07 | Negative | NA | NA | 23,66 | Negative |  |
| 164 | 185 | NA | NA | 27,8 | Negative | 36,62 | N/A | 26,47 | Positive | 1,30E+01 |
| 165 | 186 | NA | NA | 24,16 | Negative | N/A | N/A | 20,56 | Negative |  |
| 166 | 187 | NA | NA | 27,92 | Negative | 38,32 | NA | 25,72 | Positive | 4,52E+00 |
| 167 | 188 | NA | NA | 28,16 | Negative | N/A | N/A | 23,14 | Negative |  |
| 168 | 189 | NA | NA | 25,34 | Negative | N/A | N/A | 22,53 | Negative |  |
| 169 | 190 | NA | NA | 24,77 | Negative | N/A | N/A | 27,32 | Negative |  |
| 170 | 191 | NA | NA | 26,9 | Negative | N/A | N/A | 23,76 | Negative |  |
| 171 | 192 | NA | NA | 26,04 | Negative | 36,64 | N/A | 23,35 | Positive | 1,29E+01 |
| 172 | 197 | 28,31 | 38,81 | 25,33 | Positive | 26,37 | 26,9 | 23,68 | Positive | 7,72E+03 |
| 173 | 198 | 26,54 | 28,06 | 25,93 | Positive | 18,64 | 19,37 | 24,1 | Positive | 9,51E+05 |
| 174 | 199 | 19,90 | 21,23 | 27,47 | Positive | 15,28 | 16,06 | NA | Positive | 7,71E+06 |
| 175 | 200 | 24,70 | 25,71 | 32,05 | Positive | 20,15 | 21,34 | 32,08 | Positive | 3,71E+05 |
| 176 | 201 | 20,11 | 21,44 | 25,74 | Positive | 14,48 | 15,28 | 28,24 | Positive | 1,27E+07 |
| 177 | 202 | 25,74 | 26,06 | 25,40 | Positive | 20,08 | 21,13 | 24,47 | Positive | 3,88E+05 |
| 178 | 203 | 25,95 | 25,91 | 25,46 | Positive | 19,31 | 19,96 | 22,99 | Positive | 6,27E+05 |
| 179 | 204 | 24,27 | 24,42 | 25,66 | Positive | 16,36 | 17,03 | 20,12 | Positive | 3,94E+06 |
| 180 | 205 | 20,70 | 21,00 | 25,63 | Positive | 15,12 | 15,46 | 33,43 | Positive | 8,52E+06 |
| 181 | 206 | 28,13 | 27,60 | 28,14 | Positive | 20,32 | 21,59 | 35 | Positive | 3,34E+05 |
| 182 | 207 | 13,15 | 12,31 | NA | Positive | 12,26 | 13,16 | 35,37 | Positive | 5,06E+07 |
| 183 | 208 | 23,78 | 21,40 | 23,73 | Positive | 16,97 | 18,1 | 27,3 | Positive | 2,69E+06 |
| 184 | 209 | 27,36 | 27,76 | 26,02 | Positive | 20,88 | 21,74 | 26,05 | Positive | 2,36E+05 |
| 185 | 210 | 27,96 | 27,53 | 23,07 | Positive | 20,61 | 21,71 | 23,81 | Positive | 2,79E+05 |
| 186 | 211 | 27,45 | 26,96 | 26,00 | Positive | 20,53 | 21,06 | 23,7 | Positive | 2,93E+05 |
| 187 | 212 | 26,74 | 26,59 | 25,37 | Positive | 21,64 | 22,61 | 23,3 | Positive | 1,47E+05 |
| 188 | 213 | 25,05 | 24,18 | 24,01 | Positive | 16,69 | 17,27 | 19,34 | Positive | 3,20E+06 |
| 189 | 214 | 19,34 | 18,35 | 24,20 | Positive | 15,39 | 16,28 | 25,53 | Positive | 7,20E+06 |
| 190 | 215 | 25,19 | 25,17 | 25,53 | Positive | 17,37 | 17,96 | 24,07 | Positive | 2,10E+06 |
| 191 | 216 | 19,22 | 17,17 | 29,30 | Positive | 18,97 | 19,35 | 27,3 | Positive | 7,75E+05 |
| 192 | 217 | 23,60 | 22,26 | 25,45 | Positive | 18,24 | 19,31 | 25,59 | Positive | 1,22E+06 |
| 193 | 218 | 27,57 | 32,36 | 25,38 | Positive | 18 | 18,46 | 20,83 | Positive | 1,42E+06 |
| 194 | 219 | 20,16 | 20,28 | 21,97 | Positive | 12,22 | 12,79 | 29,63 | Positive | 5,19E+07 |
| 195 | 220 | 33,43 | 33,73 | 26,55 | Positive | 30,84 | 35,88 | 22,07 | Positive | 4,77E+02 |
| 196 | 221 | 20,69 | 20,91 | 23,74 | Positive | 18,36 | 20,61 | 27,32 | Positive | 1,13E+06 |
| 197 | 222 | 17,18 | 17,09 | 32,27 | Positive | 13,53 | 13,99 | NA | Positive | 2,29E+07 |
| 198 | 223 | 17,89 | 16,59 | 38,00 | Positive | 15,92 | 16,68 | 20,16 | Positive | 5,18E+06 |
| 199 | 224 | 27,23 | 26,73 | 27,92 | Positive | 23,42 | 25,29 | 26,07 | Positive | 4,85E+04 |
| 200 | 225 | 21,17 | 2111,00 | 25,12 | Positive | 18,33 | 19,11 | 23,44 | Positive | 1,15E+06 |
| 201 | 226 | 26,86 | 26,28 | 25,26 | Positive | 16,11 | 16,66 | 19,17 | Positive | 4,60E+06 |
| 202 | 227 | 29,51 | 20,05 | 26,55 | Positive | 21,07 | 21,81 | 22,57 | Positive | 2,09E+05 |
| 203 | 228 | 24,85 | 24,29 | 24,57 | Positive | 15,45 | 16,51 | 25,35 | Positive | 6,94E+06 |
| 204 | 229 | 30,98 | 28,96 | 25,80 | Positive | 26,6 | 26,99 | 25,04 | Positive | 6,69E+03 |
| 205 | 230 | 31,96 | 31,46 | 28,07 | Positive | 26,04 | 26,45 | 24,47 | Positive | 9,48E+03 |
| 206 | 231 | 26,57 | 25,87 | 25,59 | Positive | 18,46 | 19,2 | 22,31 | Positive | 1,06E+06 |
| 207 | 232 | 25,04 | 24,50 | 26,10 | Positive | 23,66 | 29,76 | 23,44 | Positive | 4,17E+04 |
| 208 | 233 | 19,53 | 28,16 | 23,26 | Positive | 10,58 | 12,16 | 24,37 | Positive | 1,44E+08 |
| 209 | 234 | 28,56 | 27,23 | 26,17 | Positive | 19,55 | 20,21 | 21,16 | Positive | 5,40E+05 |
| 210 | 235 | 25,47 | 24,22 | 24,53 | Positive | 15,11 | 15,78 | 19,01 | Positive | 8,57E+06 |
| 211 | 236 | 31,47 | 30,54 | 28,96 | Positive | 29,67 | 31,78 | 26,27 | Positive | 9,88E+02 |
| 212 | 237 | 22,06 | 22,07 | 24,72 | Positive | 15,35 | 15,8 | 18,41 | Positive | 7,38E+06 |
| 213 | 238 | 24,70 | 24,59 | 26,82 | Positive | 17,78 | 18,53 | 24,,85 | Positive | 1,63E+06 |
| 214 | 239 | 23,49 | 23,00 | 24,66 | Positive | 15,91 | 16,75 | 19,05 | Positive | 5,21E+06 |
| 215 | 240 | 20,45 | 19,80 | 23,44 | Positive | 13,74 | 14,29 | 22,16 | Positive | 2,01E+07 |
| 216 | 241 | 20,08 | 20,85 | 2,78 | Positive | 12,07 | 12,58 | 30,74 | Positive | 5,69E+07 |
| 217 | 242 | 30,77 | 31,23 | 28,91 | Positive | 20,76 | 21,57 | 29,49 | Positive | 2,54E+05 |
| 218 | 243 | 23,24 | 22,17 | 25,08 | Positive | 18,49 | 19,21 | 26,49 | Positive | 1,04E+06 |
| 219 | 244 | 31,12 | 29,68 | 27,03 | Positive | 26,71 | 27,24 | 24,36 | Positive | 6,24E+03 |
| 220 | 245 | 18,41 | 18,00 | 26,87 | Positive | 14,49 | 14,6 | 19 | Positive | 1,26E+07 |
| 221 | 246 | 27,16 | 26,34 | 27,31 | Positive | 21,3 | 21,68 | 23,14 | Positive | 1,81E+05 |
| 222 | 247 | 19,36 | 18,53 | 31,59 | Positive | 17,22 | 17,99 | 39,42 | Positive | 2,30E+06 |
| 223 | 248 | 26,71 | 25,49 | 25,71 | Positive | 18,05 | 18,69 | 20,45 | Positive | 1,37E+06 |
| 224 | 249 | 23,01 | 24,42 | 24,50 | Positive | 13,8 | 13,94 | 26,24 | Positive | 1,94E+07 |
| 225 | 250 | 28,22 | 27,50 | 25,19 | Positive | 24,78 | 24,51 | 24,07 | Positive | 2,08E+04 |
| 226 | 251 | 24,98 | 26,38 | 29,26 | Positive | 19,56 | 19,5 | 22,84 | Positive | 5,36E+05 |
| 227 | 252 | 25,58 | 26,89 | 25,53 | Positive | 14,41 | 15,03 | 22,77 | Positive | 1,33E+07 |
| 228 | 253 | 21,88 | 22,15 | 26,42 | Positive | 16,76 | 17,48 | 22,7 | Positive | 3,07E+06 |
| 229 | 254 | 26,04 | 35,55 | 28,10 | Positive | 29,57 | 29,95 | 25,2 | Positive | 1,05E+03 |
| 230 | 255 | 20,5 | 19,7 | 26,25 | Positive | 14,47 | 14,25 | 27,83 | Positive | 1,28E+07 |
| 231 | 256 | 27,62 | 28,6 | 26,25 | Positive | 18,54 | 19,22 | 22,33 | Positive | 1,01E+06 |
| 232 | 257 | 24,63 | 25,09 | 26,11 | Positive | 15,83 | 16,66 | 28,5 | Positive | 5,48E+06 |
| 233 | 258 | 19,3 | 20,4 | 23,8 | Positive | 16,7 | 16,81 | NA | Positive | 3,19E+06 |
| 234 | 259 | 23,31 | 23,65 | 28,73 | Positive | 14,53 | 15,22 | 28,48 | Positive | 1,23E+07 |
| 235 | 260 | 30,17 | NA | 26,63 | Positive | 14,76 | 15,63 | 41,73 | Positive | 1,07E+07 |
| 236 | 261 | NA | NA | 26,08 | Negative | NA | NA | 24,7 | Negative |  |
| 237 | 262 | NA | NA | 26,32 | Negative | NA | NA | 23,45 | Negative |  |
| 238 | 263 | NA | NA | 27,07 | Negative | NA | NA | 24,42 | Negative |  |
| 239 | 264 | NA | NA | 23,96 | Negative | 26,24 | 27,43 | 24,29 | Positive | 8,37E+03 |
| 240 | 265 | NA | NA | 24,79 | Negative | NA | NA | 24,07 | Negative |  |
| 241 | 266 | NA | NA | 2707 | Negative | NA | NA | 27,95 | Negative |  |
| 242 | 267 | NA | 6,95 | 27,15 | Negative | NA | NA | 24,52 | Negative |  |
| 243 | 268 | NA | 8,83 | 24,2 | Negative | NA | NA | 23,25 | Negative |  |
| 244 | 269 | NA | NA | 26,2 | Negative | NA | NA | 26,35 | Negative |  |
| 245 | 270 | 38,8 | NA | 28,9 | Negative | NA | NA | 29,36 | Negative |  |
| 246 | 271 | NA | NA | 25,96 | Negative | NA | NA | 24,24 | Negative |  |
| 247 | 272 | NA | NA | 21,5 | Negative | NA | NA | 20,11 | Negative |  |
| 248 | 273 | 40,54 | NA | 26,58 | Negative | 28,69 | 30,38 | 24,288 | Positive | 1,82E+03 |
| 249 | 274 | NA | NA | 27,21 | Negative | NA | NA | 26,92 | Negative |  |
| 250 | 275 | NA | NA | 26,16 | Negative | NA | NA | 24,82 | Negative |  |
| 251 | 276 | NA | NA | 25,06 | Negative | 31,88 | NA | 21,31 | Positive | 2,50E+02 |
| 252 | 277 | NA | NA | 23,25 | Negative | NA | NA | 23,84 | Negative |  |
| 253 | 278 | NA | NA | 25,36 | Negative | NA | NA | 25,1 | Negative |  |
| 254 | 279 | NA | NA | 28,04 | Negative | NA | NA | 22,93 | Negative |  |
| 255 | 280 | NA | NA | 24,81 | Negative | NA | NA | 23,79 | Negative |  |
| 256 | 281 | 39,27 | NA | 26,91 | Negative | NA | NA | 22,48 | Negative |  |
| 257 | 282 | NA | NA | 26,88 | Negative | NA | NA | 22,56 | Negative |  |
| 258 | 283 | NA | NA | 27,3 | Negative | NA | NA | 22,79 | Negative |  |
| 259 | 284 | NA | NA | 26,34 | Negative | NA | NA | 23,89 | Negative |  |
| 260 | 285 | NA | NA | 25,96 | Negative | NA | NA | 24,83 | Negative |  |
| 261 | 286 | NA | NA | 27,62 | Negative | NA | NA | 24,02 | Negative |  |
| 262 | 287 | NA | NA | 26,39 | Negative | NA | NA | 25,86 | Negative |  |
| 263 | 288 | NA | NA | 26,15 | Negative | NA | NA | 24,55 | Negative |  |
| 264 | 289 | NA | NA | 27,79 | Negative | NA | NA | 26,62 | Negative |  |
| 265 | 290 | NA | NA | 28,11 | Negative | NA | NA | 25,88 | Negative |  |
| 266 | 291 | NA | NA | 28,49 | Negative | NA | NA | 27,11 | Negative |  |
| 267 | 292 | NA | NA | 27,5 | Negative | NA | NA | 24,21 | Negative |  |
| 268 | 293 | NA | NA | 27,25 | Negative | NA | NA | 23,99 | Negative |  |
| 269 | 294 | NA | NA | 22,84 | Negative | 31,11 | 34,78 | 22,68 | Positive | 4,03E+02 |
| 270 | 295 | NA | NA | 27,58 | Negative | NA | NA | 26,28 | Negative |  |
| 271 | 296 | NA | NA | 28,9 | Negative | NA | NA | 26,29 | Negative |  |
| 272 | 297 | 39,31 | NA | 28,61 | Negative | NA | NA | 25,69 | Negative |  |
| 273 | 298 | NA | NA | 25,5 | Negative | NA | NA | 25,115 | Negative |  |
| 274 | 299 | NA | NA | 27,21 | Negative | NA | NA | 24,83 | Negative |  |
| 275 | 300 | NA | NA | 27,12 | Negative | NA | NA | 25,56 | Negative |  |
| 276 | 301 | NA | NA | 25,02 | Negative | 37,34 | NA | 25,29 | Positive | 8,32E+00 |
| 277 | 302 | NA | NA | 24,85 | Negative | NA | NA | 23,68 | Negative |  |
| 278 | 303 | NA | NA | 23,51 | Negative | NA | NA | 22,79 | Negative |  |
| 279 | 304 | NA | NA | 23,04 | Negative | NA | NA | 22,04 | Negative |  |
| 280 | 305 | NA | NA | 28,59 | Negative | NA | NA | 27,47 | Negative |  |
| 281 | 306 | NA | NA | 25,08 | Negative | NA | NA | 24,34 | Negative |  |
| 282 | 307 | NA | NA | 27,27 | Negative | NA | NA | 28,25 | Negative |  |
| 283 | 308 | NA | NA | 25,16 | Negative | NA | NA | 22,93 | Negative |  |
| 284 | 309 | NA | NA | 23,15 | Negative | 28,12 | 30,06 | 22,23 | Positive | 2,59E+03 |
| 285 | 310 | NA | NA | 23,5 | Negative | 34,14 | 35,43 | 24,85 | Positive | 6,11E+01 |
| 286 | 311 | NA | NA | 26,19 | Negative | NA | NA | 24,54 | Negative |  |
| 287 | 312 | NA | NA | 22,02 | Negative | NA | NA | 21,45 | Negative |  |
| 288 | 313 | NA | NA | 23,36 | Negative | 30,47 | 37,69 | 22,13 | Positive | 6,00E+02 |
| 289 | 314 | 38,85 | NA | 25,6 | Negative | 29,08 | 30,46 | 25,09 | Positive | 1,43E+03 |
| 290 | 315 | NA | NA | 24,32 | Negative | NA | NA | 23,46 | Negative |  |
| 291 | 316 | NA | NA | 25,54 | Negative | NA | NA | 24,55 | Negative |  |
| 292 | 317 | NA | NA | 25,41 | Negative | NA | NA | 25,11 | Negative |  |
| 293 | 318 | NA | NA | 26,25 | Negative | NA | NA | 26,09 | Negative |  |
| 294 | 319 | NA | NA | 25,32 | Negative | NA | NA | 27,33 | Negative |  |
| 295 | 320 | NA | NA | 22,39 | Negative | NA | NA | 21,03 | Negative |  |
| 296 | 321 | NA | NA | 23,57 | Negative | NA | NA | 22,42 | Negative |  |
| 297 | 322 | NA | NA | 28,01 | Negative | NA | NA | 27,15 | Negative |  |
| 298 | 323 | NA | NA | 27,03 | Negative | 35,58 | 40,92 | 27,76 | Positive | 2,49E+01 |
| 299 | 324 | 32,9 | NA | 24,76 | Positive | 25,85 | 26,48 | 24,62 | Positive | 1,07E+04 |
| 300 | 325 | NA | NA | 25,06 | Negative | NA | NA | 22,45 | Negative |  |
| 301 | 326 | NA | NA | 25,54 | Negative | NA | NA | 23,78 | Negative |  |
| 302 | 327 | NA | NA | 29,24 | Negative | NA | NA | 28,01 | Negative |  |
| 303 | 328 | NA | NA | 29,07 | Negative | NA | NA | 27,72 | Negative |  |
| 304 | 329 | NA | NA | 27,58 | Negative | NA | NA | 25,24 | Negative |  |
| 305 | 330 | NA | NA | 26,55 | Negative | NA | NA | 23,39 | Negative |  |
| 306 | 331 | NA | NA | 29,15 | Negative | 34,74 | NA | 26,7 | Positive | 4,20E+01 |
| 307 | 332 | NA | NA | 27,18 | Negative | NA | NA | 28,42 | Negative |  |
| 308 | 333 | NA | NA | 28,93 | Negative | 37,84 | NA | 27,9 | Positive | 6,09E+00 |
| 309 | 334 | NA | NA | 28,84 | Negative | NA | NA | 30,18 | Negative |  |
| 310 | 335 | NA | NA | 29,78 | Negative | NA | NA | 27,24 | Negative |  |
| 311 | 336 | NA | NA | 26,36 | Negative | NA | NA | 23,04 | Negative |  |
| 312 | 337 | NA | NA | 26,49 | Negative | NA | NA | 24,7 | Negative |  |
| 313 | 338 | NA | NA | 27,08 | Negative | NA | NA | 23,92 | Negative |  |
| 314 | 339 | NA | NA | 26,64 | Negative | NA | NA | 24,92 | Negative |  |
| 315 | 340 | NA | NA | 26,18 | Negative | NA | NA | 24,38 | Negative |  |
| 316 | 341 | NA | NA | 27,66 | Negative | NA | NA | 27,35 | Negative |  |
| 317 | 342 | NA | NA | 25,68 | Negative | NA | NA | 25,91 | Negative |  |
| 318 | 343 | NA | NA | 27,00 | Negative | NA | NA | 25,15 | Negative |  |
| 319 | 344 | NA | NA | 29,52 | Negative | 37,82 | NA | 30,89 | Positive | 6,17E+00 |
| 320 | 345 | NA | NA | 28,93 | Negative | NA | NA | 27,43 | Negative |  |
| 321 | 346 | NA | NA | 27,16 | Negative | NA | NA | 27,78 | Negative |  |
| 322 | 347 | NA | NA | 27,53 | Negative | NA | NA | 27,16 | Negative |  |
| 323 | 348 | NA | NA | 26,53 | Negative | NA | NA | 25,13 | Negative |  |
| 324 | 349 | NA | NA | 29,15 | Negative | NA | NA | 27,69 | Negative |  |
| 325 | 350 | NA | NA | 28,31 | Negative | NA | NA | 25,43 | Negative |  |
| 326 | 351 | NA | NA | 23,50 | Negative | 33,38 | NA | 23,34 | Positive | 9,80E+01 |
| 327 | 352 | NA | NA | 26,13 | Negative | NA | NA | 22,6 | Negative |  |
| 328 | 353 | NA | NA | 29,47 | Negative | NA | NA | 28,64 | Negative |  |
| 329 | 354 | NA | NA | 28,42 | Negative | NA | NA | 26,81 | Negative |  |
| 330 | 355 | NA | NA | 26,49 | Negative | NA | NA | 26,78 | Negative |  |
| 331 | 356 | NA | NA | 26,08 | Negative | 35,05 | 30,97 | 24,6 | Positive | 3,46E+01 |
| 332 | 357 | NA | NA | 26,84 | Negative | NA | NA | 26,03 | Negative |  |
| 333 | 358 | NA | NA | 26,62 | Negative | NA | NA | 24,93 | Negative |  |
| 334 | 359 | NA | NA | 27,19 | Negative | NA | NA | 26,08 | Negative |  |
| 335 | 360 | NA | NA | 26,15 | Negative | NA | NA | 25,35 | Negative |  |
| 336 | 361 | NA | NA | 28,28 | Negative | NA | NA | 26,41 | Negative |  |
| 337 | 362 | NA | NA | 29,11 | Negative | NA | NA | 28,12 | Negative |  |
| 338 | 363 | NA | NA | 26,51 | Negative | NA | NA | 25,38 | Negative |  |
| 339 | 364 | NA | NA | 25,67 | Negative | NA | NA | 25 | Negative |  |
| 340 | 365 | NA | NA | 28,52 | Negative | NA | NA | 30,85 | Negative |  |
| 341 | 366 | NA | NA | 27,29 | Negative | NA | NA | 26,34 | Negative |  |
| 342 | 367 | NA | NA | 26,40 | Negative | NA | NA | 27,29 | Negative |  |
| 343 | 368 | NA | NA | 25,29 | Negative | NA | NA | 23,78 | Negative |  |
| 344 | 369 | NA | NA | 25,93 | Negative | NA | NA | 23,32 | Negative |  |
| 345 | 370 | NA | NA | 27,44 | Negative | NA | NA | 26,5 | Negative |  |
| 346 | 371 | NA | NA | 26,12 | Negative | NA | NA | 24,23 | Negative |  |
| 347 | 372 | NA | NA | 25,27 | Negative | NA | NA | 24,56 | Negative |  |
| 348 | 373 | NA | NA | 24,17 | Negative | 30,56 | 31,55 | 21,89 | Positive | 5,68E+02 |
| 349 | 374 | NA | NA | 25,75 | Negative | NA | NA | 22,1 | Negative |  |
| 350 | 375 | NA | NA | 25,89 | Negative | NA | NA | 24,32 | Negative |  |
| 351 | 376 | NA | NA | 27,78 | Negative | NA | NA | 26,74 | Negative |  |
| 352 | 377 | NA | NA | 27,17 | Negative | NA | NA | 25,37 | Negative |  |
| 353 | 378 | NA | NA | 27,39 | Negative | NA | NA | 27,38 | Negative |  |
| 354 | 379 | NA | NA | 25,15 | Negative | NA | NA | 22,75 | Negative |  |
| 355 | 380 | NA | NA | 29,34 | Negative | 39,89 | NA | 26,88 | Negative |  |
| 356 | 385 | NA | NA | 26,18 | Negative | NA | NA | 30,18 | Negative |  |
| 357 | 386 | NA | NA | 26,91 | Negative | NA | NA | 24,44 | Negative |  |
| 358 | 388 | NA | NA | 27,81 | Negative | NA | NA | 24,35 | Negative |  |
| 359 | 389 | NA | NA | 28,29 | Negative | NA | NA | 26,15 | Negative |  |
| 360 | 391 | NA | NA | 29,38 | Negative | NA | NA | 26,09 | Negative |  |
| 361 | 392 | NA | NA | 27,02 | Negative | NA | NA | 24,42 | Negative |  |
| 362 | 393 | NA | NA | 26,45 | Negative | NA | NA | 24,79 | Negative |  |
| 363 | 394 | NA | NA | 26,65 | Negative | NA | NA | 25,23 | Negative |  |
| 364 | 395 | NA | NA | 26,29 | Negative | NA | NA | 24,27 | Negative |  |
| 365 | 397 | NA | NA | 27,21 | Negative | NA | NA | 21,78 | Negative |  |
| 366 | 399 | NA | NA | 23,82 | Negative | NA | NA | 19,88 | Negative |  |
| 367 | 400 | NA | NA | 28,17 | Negative | NA | NA | 22,51 | Negative |  |
| 368 | 402 | NA | NA | 26,28 | Negative | NA | NA | 22,18 | Negative |  |
| 369 | 403 | NA | NA | 27,44 | Negative | NA | NA | 22,55 | Negative |  |
| 370 | 404 | NA | NA | 29,15 | Negative | NA | NA | 26,85 | Negative |  |
| 371 | 406 | NA | NA | 25,58 | Negative | NA | NA | 31,92 | Negative |  |
| 372 | 407 | NA | NA | 27,22 | Negative | NA | NA | 22,56 | Negative |  |
| 373 | 408 | NA | NA | 23,86 | Negative | NA | NA | 18,78 | Negative |  |
| 374 | 409 | NA | NA | 27,22 | Negative | NA | NA | 21,6 | Negative |  |
| 375 | 412 | NA | NA | 28,08 | Negative | NA | NA | 22,59 | Negative |  |
| 376 | 413 | NA | NA | 26,2 | Negative | NA | NA | 21,4 | Negative |  |
| 377 | 415 | NA | NA | 30,02 | Negative | NA | NA | 24,17 | Negative |  |
| 378 | 416 | NA | NA | 26,87 | Negative | NA | NA | 23,69 | Negative |  |
| 379 | 417 | NA | NA | 25,02 | Negative | NA | NA | 21,62 | Negative |  |
| 380 | 418 | NA | NA | 26,74 | Negative | NA | NA | 21,53 | Negative |  |
| 381 | 419 | NA | NA | 26,58 | Negative | NA | NA | 20,41 | Negative |  |
| 382 | 420 | NA | NA | 26,11 | Negative | NA | NA | 20,19 | Negative |  |
| 383 | 421 | NA | NA | 25,8 | Negative | NA | NA | 20,35 | Negative |  |
| 384 | 422 | NA | NA | 25,03 | Negative | NA | NA | 19,88 | Negative |  |
| 385 | 423 | NA | NA | 27,28 | Negative | NA | NA | 21,4 | Negative |  |
| 386 | 424 | NA | NA | 28,18 | Negative | NA | NA | 28,07 | Negative |  |
| 387 | 425 | NA | NA | 28,72 | Negative | NA | NA | 24,25 | Negative |  |
| 388 | 426 | NA | NA | 27,71 | Negative | NA | NA | 25,35 | Negative |  |
