# Supplementary material for: Fast, cheap and sensitive: Homogenizer-based RNA extraction free method for SARS-CoV-2 detection by RT-qPCR
Source: Front Cell Infect Microbiol. 2023 Mar 9;13:1074953. doi: 10.3389/fcimb.2023.1074953 (PMC10035754; doi:10.3389/fcimb.2023.1074953)
Supplement: Supplementary file 2 [file Table_2.docx]

**Supplementary Table 2. Ct values and viral load for the 25 false negative samples for SARS-CoV-2 by the RNA extraction free homogenization method compared to column RNA extraction (N/A means "not amplified").**

|  | | Homogenization method | | | | Column RNA extraction | | | | |
| --- | --- | --- | --- | --- | --- | --- | --- | --- | --- | --- |
| N | **Code** | **N1 Ct** | **N2 Ct** | **RP Ct** | **Result** | **N1 Ct** | **N2 Ct** | **RP Ct** | **Result** | **Viral Load (copies/uL)** |
| 1 | 162 | NA | NA | 26,08 | Negative | 36,9 | 37,02 | 22,34 | Positive | 1,09 x 10^1^ |
| 2 | 164 | NA | NA | 27,05 | Negative | 34,01 | 37,21 | 28,12 | Positive | 6,62 x 10^1^ |
| 3 | 165 | NA | NA | 24,65 | Negative | 37,58 | N/A | 20,72 | Positive | 7,17 |
| 4 | 170 | NA | NA | 27,03 | Negative | 33,64 | N/A | 24,59 | Positive | 8,34 x 10^1^ |
| 5 | 181 | NA | NA | 27,13 | Negative | 38,03 | N/A | 24,9 | Positive | 5,41 |
| 6 | 182 | NA | NA | 24,04 | Negative | 33,53 | NA | 22,26 | Positive | 8,93 x 10^1^ |
| 7 | 185 | NA | NA | 27,8 | Negative | 36,62 | N/A | 26,47 | Positive | 1,30 x 10^1^ |
| 8 | 187 | NA | NA | 27,92 | Negative | 38,32 | NA | 25,72 | Positive | 4,52 |
| 9 | 192 | NA | NA | 26,04 | Negative | 36,64 | N/A | 23,35 | Positive | 1,29 x 10^1^ |
| 10 | **264** | **NA** | **NA** | **23,96** | **Negative** | **26,24** | **27,43** | **24,29** | **Positive** | **8,37 x 10^3^** |
| 11 | **273** | **NA** | **NA** | **26,58** | **Negative** | **28,69** | **30,38** | **24,28** | **Positive** | **1,82 x 10^3^** |
| 12 | 276 | NA | NA | 25,06 | Negative | 31,88 | NA | 21,31 | Positive | 2,50 x 10^2^ |
| 13 | 294 | NA | NA | 22,84 | Negative | 31,11 | 34,78 | 22,68 | Positive | 4,03 x 10^2^ |
| 14 | 301 | NA | NA | 25,02 | Negative | 37,34 | NA | 25,29 | Positive | 8,32 |
| 15 | **309** | **NA** | **NA** | **23,15** | **Negative** | **28,12** | **30,06** | **22,23** | **Positive** | **2,59 x 10^3^** |
| 16 | 310 | NA | NA | 23,5 | Negative | 34,14 | 35,43 | 24,85 | Positive | 6,11 x 10^1^ |
| 17 | 313 | NA | NA | 23,36 | Negative | 30,47 | 37,69 | 22,13 | Positive | 6,00 x 10^2^ |
| 18 | **314** | **NA** | **NA** | **25,6** | **Negative** | **29,08** | **30,46** | **25,09** | **Positive** | **1,43 x 10^3^** |
| 19 | 323 | NA | NA | 27,03 | Negative | 35,58 | 40,92 | 27,76 | Positive | 2,49 x 10^1^ |
| 20 | 331 | NA | NA | 29,15 | Negative | 34,74 | NA | 26,7 | Positive | 4,20 x 10^1^ |
| 21 | 333 | NA | NA | 28,93 | Negative | 37,84 | NA | 27,9 | Positive | 6,09 |
| 22 | 344 | NA | NA | 29,52 | Negative | 37,82 | NA | 30,89 | Positive | 6,17 |
| 23 | 351 | NA | NA | 23,50 | Negative | 33,38 | NA | 23,34 | Positive | 9,80 x 10^1^ |
| 24 | 356 | NA | NA | 26,08 | Negative | 35,05 | 30,97 | 24,6 | Positive | 3,46 x 10^1^ |
| 25 | 373 | NA | NA | 24,17 | Negative | 30,56 | 31,55 | 21,89 | Positive | 5,68 x 10^2^ |
